# Enhanced thermal management of mats and yarns from polystyrene fibers through incorporation of exfoliated graphite†

**DOI:** 10.1039/d4ma01162g

**Published:** 2025-02-17

**Authors:** Madhurima Das, Joanna Knapczyk-Korczak, Ahmadreza Moradi, Waldemar Pichór, Urszula Stachewicz

**Affiliations:** a Faculty of Metals Engineering and Industrial Computer Science, AGH University of Krakow Krakow 30-059 Poland ustachew@agh.edu.pl; b Faculty of Materials Science and Ceramics, AGH University of Krakow al. A. Mickiewicza 30 30-059 Kraków Poland

## Abstract

The energy crisis, driven by modern electronics and global warming from population growth, underscores the need for advanced textiles to regulate thermal environments. Researchers stress the need to improve high-performance polymer mats with enhanced thermal conductivity. This report delves into the morphological, mechanical, and thermal properties of exfoliated graphite (EG) when incorporated into polystyrene (PS) fiber mats and yarns through blend electrospinning. The incorporation of EG inside the fibers allowed us to obtain approximately twofold improvement in maximum stress and toughness compared to pristine PS mats. Thermal camera measurement showed significant improvement in heat transport for PS–EG fibers. The heating test showed a temperature increase of ∼2.5 °C for an EG-loaded PS mat, and in the case of a resistance wire coated with a PS fiber yarn, the increase reached 17 °C. The incorporation of EG into electrospun mats enables the recovery of more energy in the form of heat by enhancing the heating of the sample through infrared radiation. The temperature increased by 2 °C for PS and by 27 °C for PS–EG, respectively. The obtained results exhibit a great potential for the application of electrospun hybrid systems with EG in further advancement in the field of next-generation thermal management.

## Abstract 2

The incorporation of thermally conductive exfoliated graphite into polystyrene fibers significantly enhances their thermal conductivity. This modification in the yarn structure optimizes thermal management by enabling effective heat transfer.

## Graphical abstract

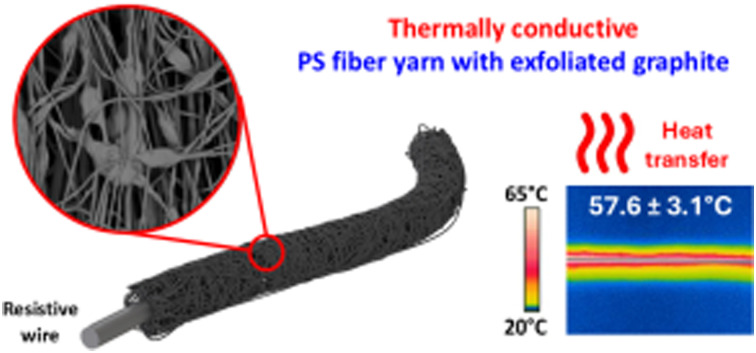

## 1. Introduction

As we navigate the transition to the 5G era and grapple with the challenges of energy consumption, device efficacy, and lifetime, it is imperative to prioritize innovations of novel materials that are not only suitable for energy harvesting^1–3^ but also to dissipate generated heat to enhance electronic device performance for the creation of sustainable society.^4^ Engineered polymeric materials have been attracting diverse research interest for the last several years owing to their lightweight,^5^ flexibility,^6^ ease of processibility,^7^ and tunable thermal conductivity.^8^ However, the intrinsic poor thermal conductivity of polymers poses a challenge in meeting the high demand of longitudinal heat conduction and dissipation for real field implementation.^9^ The synthesis of advanced polymers with high tunable thermal conductivity^10,11^ or the introduction of different thermally conductive fillers^12–15^ to polymers are the two most well-adopted strategies to fabricate high-performance thermally conductive materials. However, the geometry of fillers, their inhomogeneous distribution in a polymer matrix, and interactions between fillers and polymer can significantly increase the internal thermal resistance^16–19^ and heat scattering along with poor mechanical attributes due to different levels of filler loading.^20–22^

Electrospinning represents a unique approach to achieving a homogeneous distribution and directional alignment of fillers or functional agents within polymers,^23–28^ leading to the creation of flexible, porous polymeric structures with a high surface area.^29–31^ However, the porous architecture of the electrospun mat and its low thermal conductivity^32^ are the main drawbacks to improving the heat transfer process throughout the porous architecture.^33,34^ Advanced manufacturing processes, such as yarn electrospinning or hot-pressing technique allow for an adjustment in the porosity between the fibers and enhance mechanical properties.^4,35^ The simple manufacturing of textiles from yarn is another added advantage of this methodology, paving the way for widespread application zones.^36–39^

Different carbon-based nanofillers derived from graphite and associated composites^40–43^ have risen as a promising option, aiming to revamp the thermal conductivity and thermal management efficacy of the system.^44–46^ The reasonable cost, high aspect ratio,^47,48^ high Young's modulus (∼1 TPa),^49^ and outstanding in-plane thermal conductivity up to 500–5400 W m^−1^ K^−1^ depending on the nature of carbon nanofillers^50–52^ are the main reasons for its widespread applicability in the thermal management arena. Among different forms of carbon fillers, exfoliated graphite (EG) is considered as one of the potential light-weight, low cost carbonaceous additives to revamp the thermal conductivity of the composite along with simplified composite preparation process.^43,53–55^ For example, Xiao *et al*. achieved ∼6.2 times increment in thermal conductivity after adding 20 wt% graphene nanoplatelet in CNT/polyvinylidene fluoride (PVDF) composite.^56^ Zhu *et al*. reported excellent thermal conductivity and tensile strength of rGO/polyimide (PI) film, which reached 1467 ± 55 W m^−1^ K^−1^ and 142 ± 11 MPa, respectively.^57^

Among various polymers, the polystyrene (PS) stands out as one of the most promising multifunctional materials due to its affordability, lightweight nature, ease of processing, durability, and resistance to humidity.^58–60^ Despite such unique advantages, inferior thermal conductivity and limited mechanical properties depending on the molecular weight of PS or the presence of co-polymer restrict its versatile utilization in thermal management applications.^61–63^ The incorporation of carbon filler can effectively improve the thermal stability, tensile strength, and Young modulus of the PS mat, as observed by Abdelhady *et al.* in an exfoliated graphite/PS composite system.^64^ Moreover, PS is an excellent electrical insulator; however, adding graphite fillers increases its electrical conductivity.^65,66^

**Fig. 1:**
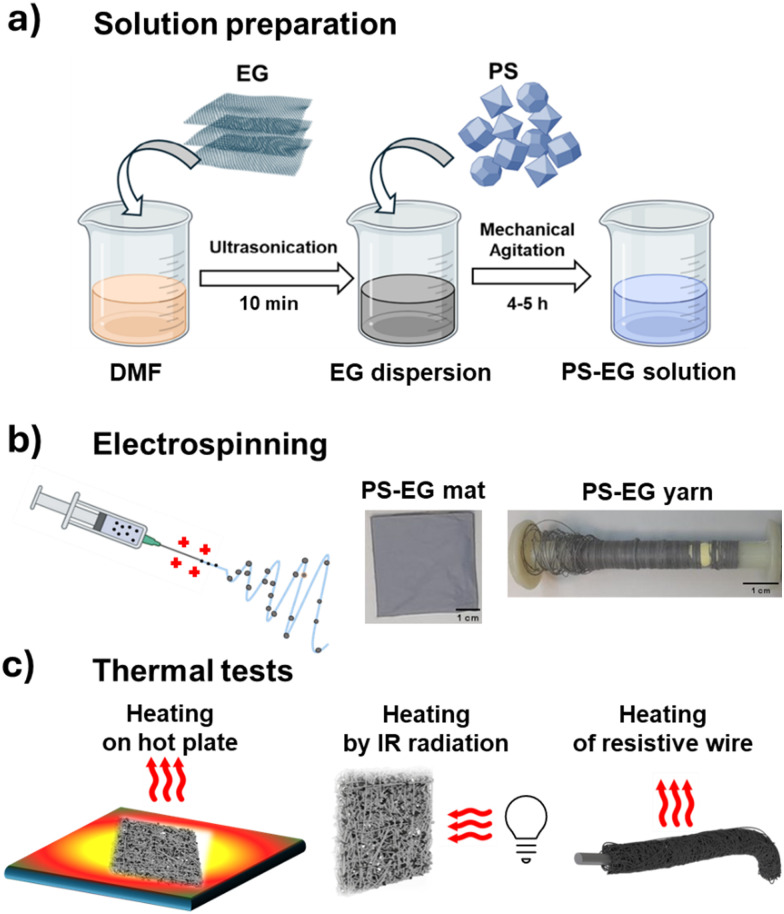
The concept of the article includes (a) manufacturing protocols for solution preparation, (b) material production, and (c) measurements of heat transfer characteristics of fiber mats and yarns.

Building on these insights, an endeavor has been made to incorporate EG into PS to enhance the mechanical and thermal properties of insulating PS through blend electrospinning. We explore the impact of EG as fillers on the mechanical and thermal performance of PS fiber mats and yarns. Various characterization techniques were employed to investigate the developed composite material's morphology, chemical composition, and mechanical and thermal properties. By adjusting the concentration of EG, we verified the impact of carbon fillers on the microstructure, mechanical and thermophysical properties of the electrospun PS mat. We managed to improve the heat transfer of EG-loaded PS fibers, which was confirmed *via* IR thermography and the measurements of the thermal conductivity coefficient (*λ*). A comprehensive concept of this article with synthesis process, macro-architecture of the material, and thermal management measurement set up for fiber mat and yarn is represented in Fig. 1. The fibrous materials innovated in this study hold promise for utilization as a layer or coating in heat dissipation, addressing a range of thermal management needs across diverse applications.

## 2. Results and discussion

### 2.1. Morphology of electrospun fibers

**Fig. 2:**
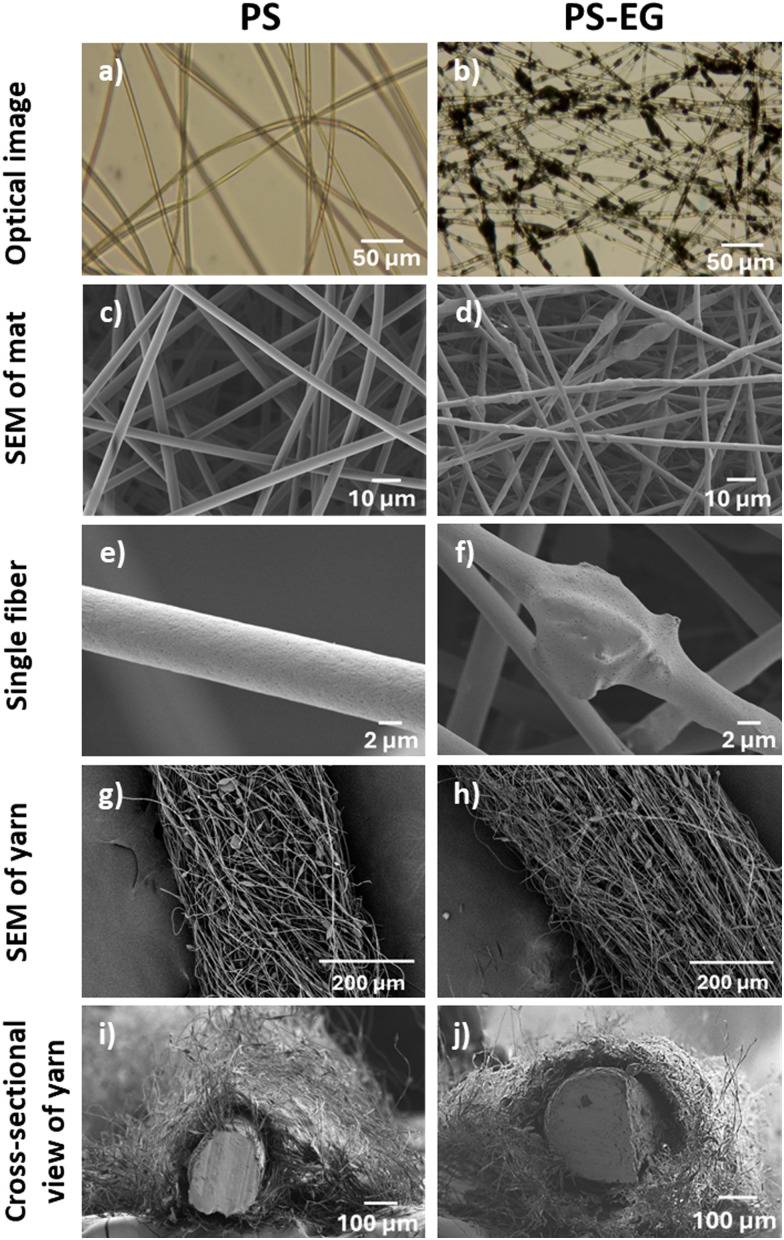
Morphology of PS and PS–EG fibers in mats and coatings on the resistive wire (yarn). (a) and (b) Optical images with EG distribution in fibers, (c)–(f) SEM images of fiber in mats with the magnified view for the single fibers, (g)–(j) SEM images of top view and cross-sectional view of fibers in yarns.

We have successfully created hybrid fibers combining PS and EG, leveraging on EG's renowned high thermal conductivity.^67^ The PS fiber mat exhibited a bead-free network, evident in both optical and SEM micrographs, see Fig. 2(a), (c) and (e). However, after introducing EG into the PS fibrous network, a distinct agglomerate-on-string-like structure emerged, see Fig. 2(b) and (d). The loading of EG flakes caused the segregation of EG particles within the PS fibers, what is clearly visible as a dark spot in the optical images. The agglomerated flake-like architecture of EG was revealed in Fig. S1 (ESI†). The SEM micrograph reveals an uneven and rough surface, indicating the agglomeration of EG sheets. The magnified view of EG-loaded single PS fibers illustrates the presence of EG flakes within the fibers accompanied by small agglomerations in the single fiber, see Fig. 2(f). We employed blend electrospinning of PS and EG dispersion to obtain PS–EG fibers. The presence of EG at the surface and within the fiber is associated with random distribution of EG during blend electrospinning process. The random distribution of thermally conducting fillers during blend electrospinning agrees with the previous studies.^4,22,68^ The calculated average diameter of pristine PS fibers was 3.52 ± 0.46 μm. However, when EG was introduced, the fiber diameter decreased to 2.84 ± 0.34 μm, see Fig. S2 (ESI†). Notably, the fiber diameter decreased with loading of EG due to an increment in the conductivity of PS–EG solutions from 0.175 ± 0.003 to 0.247 ± 0.001 S cm^−1^, see Fig. S2b in ESI.† This reduction is due to the presence of more charges at the surface of the jet, causing repulsion of the charge within the jet, which leads to extended jet elongation during electrospinning.^69–71^

### 2.2. Electrospun EG-loaded PS fiber mat

**Fig. 3:**
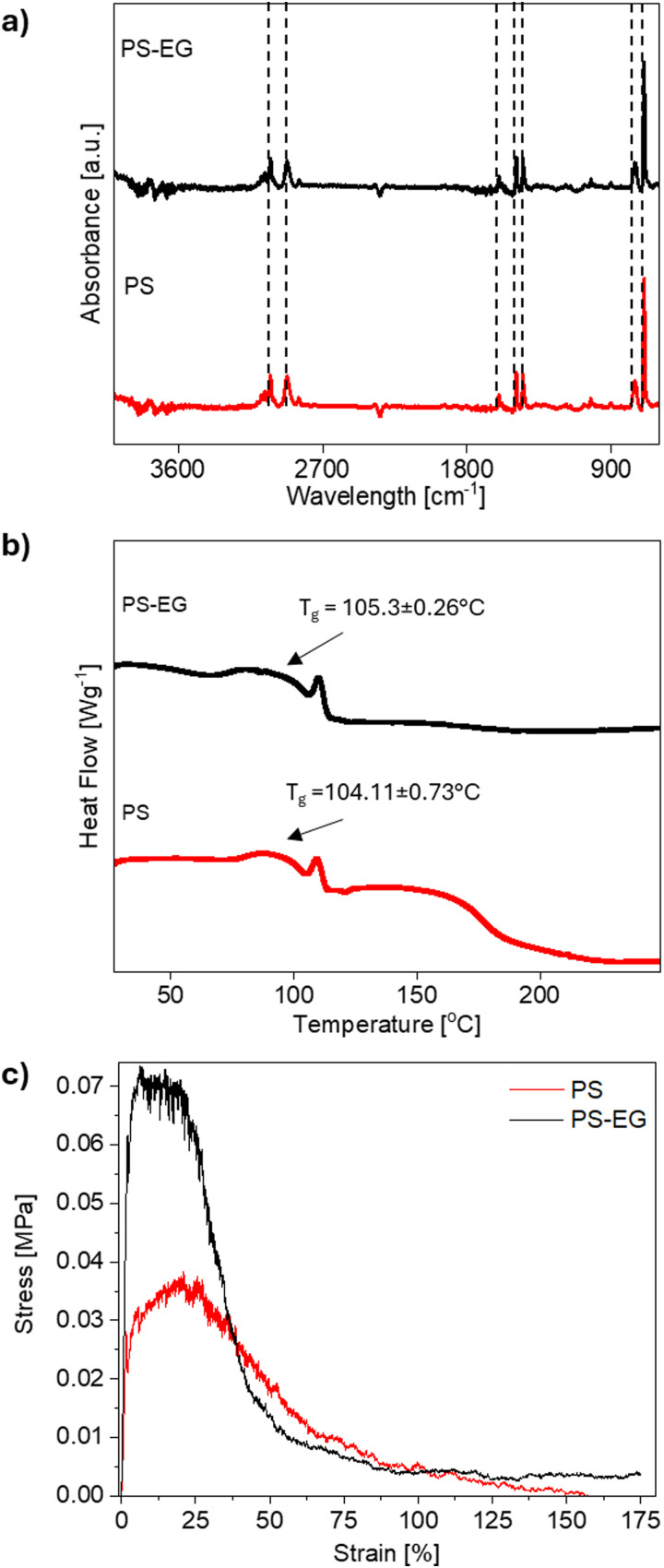
(a) The FTIR spectra, (b) DSC diagram, and (c) representative stress–strain curves for PS and PS–EG fiber mat.

Distinct FTIR peaks, see Fig. 3(a) were identified for pristine PS at 3024 corresponding to aromatic –C–H stretching vibrations, 2922 cm^−1^ for asymmetrical –CH_2_ stretching vibrations, and 1600, 1492, and 1450 cm^−1^ for C=C skeletal stretching in the aromatic ring of PS respectively.^32,72,73^ The peaks at 753 and 697 cm^−1^ were attributed to C–H out-of-plane bending vibrations of PS. The similar FTIR peak positions of PS and PS–EG mat indicate a homogeneous blending of EG particles and PS within the electrospun fiber mat. This suggests that the incorporation of EG does not significantly alter the chemical composition or structure of the electrospun fiber mat. Further, the DSC diagram of PS–EG resembles that of the pristine PS, as shown in Fig. 3(b) and Fig. S3 (ESI†). The calculated glass transition temperatures (*T*_g_) for PS, and PS–EG were 104.11 ± 0.26 and 105.3 ± 0.26 °C, respectively. The *T*_g_ value observed for PS aligns with previous reports.^30^ Moreover, the DSC results confirmed the amorphous structure of PS, see Fig. S3 in the ESI.†

Notably, the combination of EG with PS *via* blend electrospinning results in an increase in the mechanical properties of the mat compared to pristine PS, see Fig. 3(c). The maximum tensile stress of PS appears at 0.03 ± 0.01 MPa, while PS–EG mat increases to 0.07 ± 0.01 MPa. The strain at the maximum stress of tested samples was around 17%, and once this value was exceeded, there was a sharp drop in the stress. The observed maximum stress for randomly oriented PS fibers well agrees with our previous reports^74,75^ and literature data, where Yoon *et al*. observed the maximum tensile strength of 0.4 MPa.^76^ The observed result suggested that the presence of carbon fillers exhibits a reinforcement effect in the fibrous architecture through load transfer mechanism during mechanical stretching.^4^ The decrement in fiber diameter in EG loaded fibers possess high molecular rearrangement, leading to an increase in the stiffness of the fiber mat.^22^ It is evident that the PS–EG sample demonstrated higher mechanical strength compared to the PS, see Fig. S4 and Table S1 (ESI†). Previous observations by light microscopy and SEM have confirmed the formation of EG agglomerates in the fiber. The agglomeration of EG flakes can act as a stress propagation center and cause the molecules to interact more with each other rather than with the polymer matrix.^22^ However, the enhanced connection between EG and interfacial interaction with polymer is responsible for such observation.^77,78^

### 2.3. Thermal conductivity and management evaluation

Upon analyzing the temperature *versus* time curve of EG-loaded PS fiber mats, we noticed a significant contrast in temperature variation over time between the pristine PS and the PS–EG fiber mat. The thermal conductivity measurements (*λ*) were conducted in the through-plane direction of the mats and showed *λ* values of 0.031 and 0.030 W m^−1^ K^−1^ for PS and PS–EG, respectively. Obtained values are similar to our previous study where *λ* for PS and TPU-PS double-shell hollow fibers reached 0.032 W m^−1^ K^−1^ for both samples.^32^ The drawback of accurate measurement of thermal conductivity for porous 3D architecture has already been highlighted by Munoz Codorníu *et al.*^79^ The small change in *λ* value between PS and PS–EG is strongly linked to the porous network architecture of the mat, which results in dominant convective heat transport through the membrane rather than conduction during the measurement. The heat scattering at the void space filled with air in the network fibrous architecture is another reason for the low thermal conductivity of the composite. The small change in the thermal conductivity value of fiber and its mat was previously reported.^80,81^ However, Li *et al.*^82^ demonstrated an enhanced heat transfer phenomenon attributed to the presence of exfoliated graphene in epoxy nanocomposites.

**Fig. 4:**
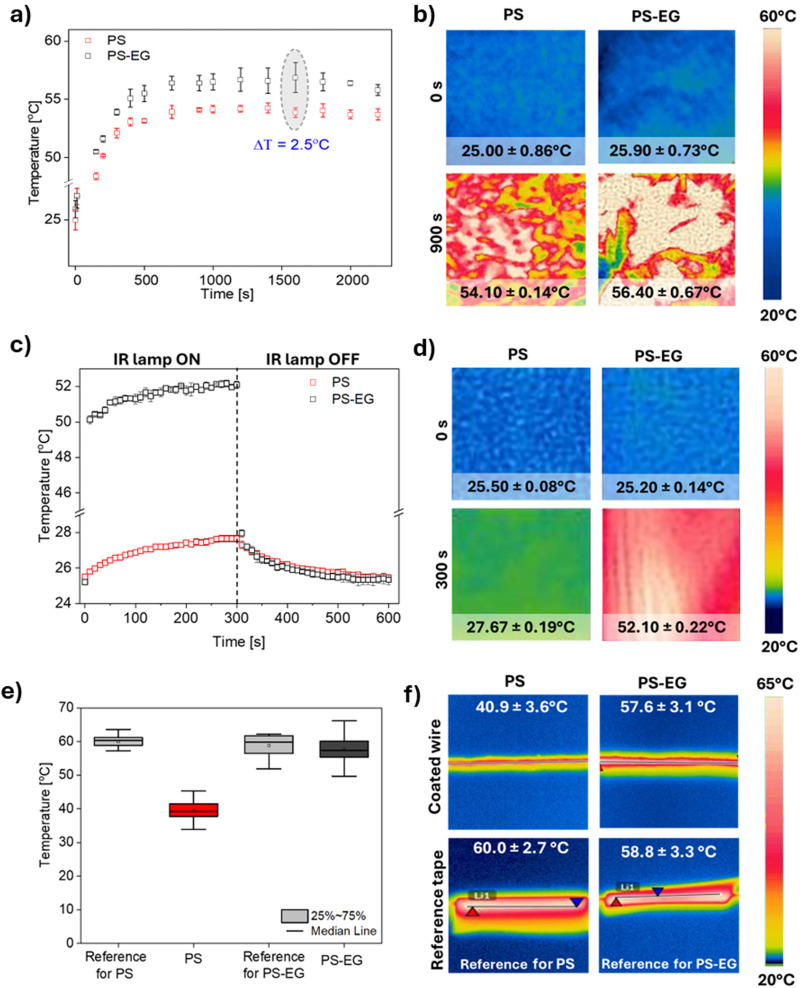
(a) Heating curves for PS and PS–EG mats, and (b) the thermal camera images of samples from heating plate measurement. (c) Heating and cooling curves for PS and PS–EG mats, and (d) the thermal camera images of samples from IR lamp measurement. (e) Heating the resistive wire coated by PS and PS–EG and the reference black tape with known emissivity (*ε* = 0.96), and (f) the thermal camera images of samples during measurement.

Here, thermal camera investigation confirmed the effect of EG flakes on the heat transfer of the PS mats. Prior to the transient thermal measurement, we measured the thickness of each fiber mat, as the thickness of any material plays a pivotal role in heat transfer attributes to compare the results. The thickness of PS and PS–EG mats were similar and reached 0.536 ± 0.028 and 0.660 ± 0.020 mm, respectively. The surface temperature changes of the fiber mats were monitored over time using a thermal camera. All fiber mats reached thermal equilibrium after 500 s. Notably, the PS–EG fiber mat exhibited a ∼2.5 °C higher surface temperature than the PS mat at thermal equilibrium, Fig. 4(a). A faster color change was evident for the PS–EG fiber mat compared to PS, as depicted in Fig. 4(b). The observed thermal camera result signifies the improved heat conduction of the PS–EG mat compared to the pristine PS mat. The random arrangements of EG sheets and agglomerates are observed at Fig. 2(b). The presence of fillers and their agglomerates in fibrous architecture is responsible to create thermal conduction pathways throughout the membrane.^22^ The heat transport phenomenon through EG agglomerates in fibrous architecture depends on various complex factors, such as, the particle size of EG, extent of EG agglomeration, and the orientation of EG sheets. The scattering of heat wave and air gap between porous architecture in fiber membrane impedes the heat transfer process, though porous architecture is imperative in personal thermal management for facile air and moisture transport. The proposed fiber mats demonstrate a comparable temperature difference to the reported literature.^83,84^

The thermal test using IR lamp heating allows us to evaluate how electrospun mats absorb heat when it is transferred *via* radiation rather than conduction, as in the heating plate test. We observed that fibers containing EG accumulate significantly more energy, as they are opaque to light, unlike pristine PS, which is relatively transparent. The mat temperature was increased ∼107% for PS–EG mat and only ∼9% for pristine PS. Adding EG alters the fiber structure, making the PS–EG composite darker and more effective in absorbing infrared radiation. This behavior is due to the photothermal effect, where EG-containing fibers convert absorbed light into heat, enhancing energy retention compared to pure PS fibers.^85,86^ We observed similar behavior in nature. The polar bear (Ursus maritimus) has unique thermoregulatory adaptations that enable it to survive in extremely low temperatures. The transparent structure of its fur reduces heat loss by scattering and reflecting heat, allowing the efficient transfer of solar energy to the bear's body.^87^ Beneath this fur, the bear's black skin further aids in heat retention by efficiently absorbing solar radiation.^88–90^

### 2.4. Wire coated with electrospun EG loaded PS yarn

As indicated by the performance of PS–EG mat, we continued the investigation for yarns. Further, we aimed to understand how the porous structure of the fiber mat transforms into a denser architecture of yarns when infused with EG. Such a shift potentially enhances the material's ability to conduct heat, presenting promising prospects for applications requiring effective thermal management. A resistive wire coating strategy was employed to assess the impact of EG-loading on the heat transfer characteristics of PS and PS–EG yarns when coated on a resistive wire, which was used here as a heating element to evaluate the thermal performance of the samples.^4^Fig. 2(g)–(j) depicts the apparent surface features and cross-section image of electrospun PS and PS–EG fibers coated on the resistive wire. The average thickness of PS and PS–EG yarns coated on wires was approximately 358 ± 47 μm and 481 ± 44 μm, respectively. The produced yarns covered the resistive wire, which was evident from the cross-section images of the samples, revealing a good adhesion between the resistive wire and fibers. This phenomenon facilitates the heat transfer between the wire and the fibers. The surface morphology of the fibers in both resistive wires coated with PS and PS–EG samples comprises bead-on-string-like features, see Fig. S5a and b (ESI†). The average fiber diameter of PS and PS–EG yarns was about 1.63 ± 0.25 μm and 1.22 ± 0.32 μm, respectively, while the average bead diameters were 7.92 ± 1.97 μm and 8.04 ± 3.39 μm. The fiber diameter of PS–EG samples decreased after the introduction of EG because of an increase in the solution conductivity, as we discussed before, see Fig. S5c (ESI†). The average diameter of beads in the fiber structure of the samples is higher for PS–EG samples due to the segregation of EG flakes during stretching under an applied electric field, see Fig. S5d (ESI†). The wrinkled surface morphology of the fibers and beads was observed in both PS and PS–EG samples. The low-humid atmosphere can cause thinning and instability of jets along with delayed solidification of the polymer jet, resulting in the formation of bead-on-string-like morphology with wrinkled surface.^91,92^ The mechanical properties of the yarns were not determined, as they were largely dependent on the strength of the resistive wire.

Thermal camera measurements were conducted to further verify the heat conduction capacity of PS–EG yarns. The applied current to the resistive wires was adjusted to control the surface temperature of the reference black tape, around 60 °C, monitored by the thermal camera. As depicted in Fig. 4(e) and (f), temperature diagrams, along with the thermal camera images of PS and PS–EG coating yarns, clearly indicate the increase in the surface temperature of the fibers after introducing the EG into the PS fibers. The average surface temperature of the resistive wire-coated PS sample was recorded as 40.9 ± 3.6 °C, while the PS–EG samples showed a higher average surface temperature of 57.6 ± 3.1 °C after reaching thermal equilibrium. We observed an approximately 17 °C increase in the surface temperature of the PS–EG sample compared to pristine PS, highlighting the excellent enhancement of the heat conduction in the composite fiber.

The compact architecture of the PS–EG yarn, characterized by an interlinked fibrous structure, creates an extended and unidirectional thermally conductive pathway through the fibrous architecture. This compact fiber architecture in coated fibers enhances through-plane thermal conductivity of the system, as mentioned previously.^4^ The observed temperature difference between PS and PS–EG samples was notably higher compared to PS and PS–EG mats, mainly attributed to the reduced porosity and enhanced interlinked connections between fibers in the yarn structure.^4^ The evaluated porosity of PS and PS–EG mats were 53.09 ± 5.69%, and 47.72 ± 4.22%, while for PS, and PS–EG yarns coated resistive wire were 6.79 ± 4.66%, and 4.99 ± 2.01% respectively. The reduced in porosity of yarn architecture compared to membrane is attributed to large temperature difference between PS and PS–EG yarn samples compared to PS and PS–EG mats, leading to enhanced interlinked connections between fibers in the yarn structure. The air gap between open pores in the mats acts as a thermal insulator, hindering the heat transfer phenomenon and leading to a decrease in thermal conductivity.^93,94^ Conversely, the effective intricate connection between fibers facilitates thermal transport through the yarns. Therefore, the proposed fabric can be employed as a cooling fabric for personal thermal management. Modification strategies provide an excellent approach to enhancing the thermal conductivity of such textile.^95,96^

**Fig. 5:**
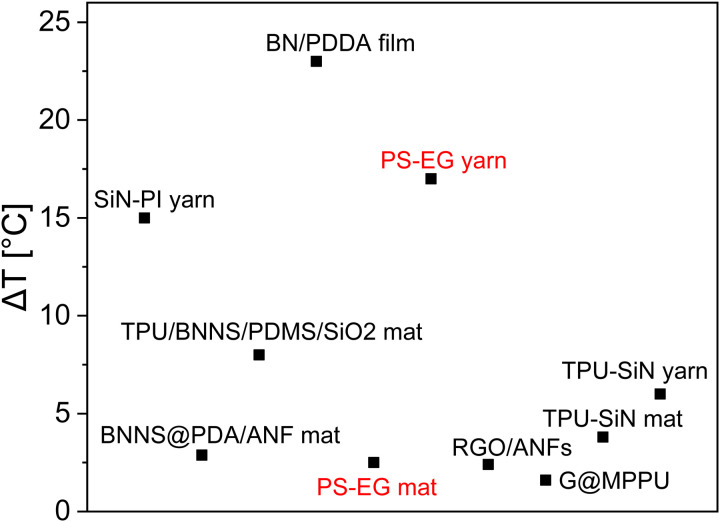
The comparison of the thermal performance of our work with recently published literature,^4,22,97–101^ where Δ*T* means thermal improvement of composite fiber compared to either pristine polymer, cotton or other reference material.

Furthermore, we have compared our results with other literature reports to highlight the improvement in thermal properties of various thermally conductive composite mats and resistive wire-coated yarns relative to pristine polymer (improvement degree, Δ*T*). In Fig. 5 we demonstrate the improvement degree (Δ*T* = 17 °C) of our fabricated PS–EG yarn with 480 μm thickness is better with respect to SiN-polyimide (PI) yarn (Δ*T* = 15 °C) with 696 μm thickness,^4^ boron nitride nanosheet (BNNS)/polydopamine (PDA)/aramide nanofiber (ANF) (Δ*T* = 2.88 °C within 15 s)^97^ and comparable with other reported literature.^22,98–101^ The improvement degree of our prepared PS–EG fiber mat (Δ*T* ∼ 2.5 °C) is comparable to the BNNS/PDA/ANF composite. The thickness of materials plays a key role in improving the heat transfer process, but most of the literature fails to report the thickness of materials. The observed comparative results highlights the potential of EG-coated textiles for enhanced heat transport.

### 2.5. Potential application with future perspective

In this study, we demonstrated the enhanced mechanical and thermal management properties of electrospun EG modified PS fiber mats and coating yarns. Thanks to their improved heat transfer capabilities and reliable mechanical properties, the PS–EG fibers will be a potential candidate as a cooling textile for human bodies and electronic devices. The resistive wire-coated yarn can also be utilized to develop advanced smart heating textiles for healthcare applications. The efficient heat transfer from the resistive wire to the PS–EG yarn surface ensures localized heating while providing extra protection to the skin from direct contact with the resistive wire.

## 3. Conclusion

As the energy crisis and global warming are driven by the increasing power demands of electronics used by a rapidly growing population, we developed hybrid high-performance mats and yarns for advanced textiles with improved thermal conductivity. We successfully created a composite fiber with exfoliated graphite to enhance the thermal conductivity of pristine PS fibers in the form of mats and yarns. Incorporating EG in the PS fiber mat radically enhances the mechanical strength and toughness of the hybrid fibers. Moreover, the interconnected distribution of EG within the electrospun composite fibers and yarns provides efficient thermal conduction pathways. Therefore, a resistive wire coated by PS–EG yarn led to a significant increment of ∼17 °C in the surface temperature in comparison to an ∼2.5 °C increase for PS–EG mat with respect to pristine PS fibers. The PS–EG sample exhibited a notable temperature increase under IR radiation by 27 °C, suggesting its potential to accumulate energy from the surrounding environment, in comparison to PS (∼2 °C). The future perspective of the fabricated material is also highlighted. The developed EG-loaded fiber mat and yarn hold great promise for diverse thermal management applications.

## 4. Experimental section

### 4.1. Materials and electrospinning

Polystyrene (PS, *M*_w_ = 350 000 g mol^−1^, Sigma Aldrich, USA) and exfoliated graphite flakes (EG, size of ∼5 μm, SGL Carbon, Germany) were utilized for the electrospinning of composite fibers. The PS with 25 wt% was dissolved in dimethylformamide (DMF Sigma Aldrich, UK). The blend of PS with EG was prepared by adding PS to EG, which had previously been dispersed in DMF by 10 min of ultrasonic bath (EMAG, Emmi-E20, Germany). The concentration of EG was 20 wt% by weight of PS, and this was the maximum value of EG which give us a spinnable solution. Then, solutions were stirred for 4 h at a magnetic stirrer (RCT basic, IKA, Germany) with a rotation speed of 400 rpm at 22 °C.

The production of electrospun fibers was performed using an electrospinning apparatus (NanoFiber Electrospinning – ESVY-100, MicroNano Tools, Canada) with the climate control set at *T* = 25 °C and RH = 40%. The applied voltage of 11 kV was applied to the stainless-steel nozzle with an outer diameter (OD) of 0.8 mm and an inner diameter (ID) of 0.5 mm, set at a distance of 20 cm to the collector, which was covered with Al foil and its rotation speed was set at 10 rpm. The produced fibers were marked as PS, and PS–EG (20 wt% of EG). The polymer solution flow rate was 1.5 ml h^−1^, and the deposition time of randomly oriented fibers took 2 h.

The coating yarns were produced using electrospinning equipment with yarn module at RH = 19 ± 3% and *T* = 25 ± 3 °C with the resistive wire as a core (resistance wire, RD 100/0.2 Block, Germany) coated with the PS and PS–EG fibers. The applied positive and negative voltage to both nozzles was 8 kV for PS and 11 kV for PS–EG samples. The distance between the nozzles to the vortex collector was 17–18 cm. The flow rate was 1.8 ml h^−1^, and the rotation speeds of the vortex and collecting mandrel were 200–250 and 5–7 rpm, respectively.

### 4.2. Material characterization

The fibrous mats’ surface and cross-section image of resistive wire-coated yarn was investigated using SEM (Merlin Gemini II, ZEISS, Germany) at 3 kV accelerating voltage, 110 pA current, and a 4–9 mm working distance. Prior to SEM imaging, the fibrous mats were coated with 8 nm Au using a sputter coater (Q150RS, Quorum Technologies, UK). The fibers’ diameter was measured from SEM micrographs using ImageJ software (version 1.51, Fiji, USA), and the average fiber diameter (*D*_f_) was calculated from 100 measurements. The surface morphology of coating yarns was captured by Phenom ProX Desktop SEM system (Thermo Fisher Scientific, Waltham, MA, USA). The samples were prepared using the freeze-fracture method in liquid nitrogen to analyze the cross-section view of the yarns. The yarn diameter was measured from randomly selected different positions of the yarns.

The porosity of PS and composite mats was evaluated using gravimetric measurement method,^102^ as described in the eqn (1):1
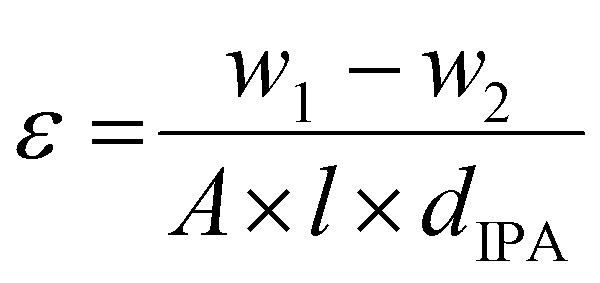
where, *w*_1_ and *w*_2_ are the weight (g) of wet and dry mat/yarn, *A* is the area of the mat/yarn (cm^2^), *l* is the mat/yarn thickness (cm), *d*_IPA_ is the density of IPA (0.786 g cm^−3^). For porosity measurement, a random piece of mat/yarn with definite dimension was immersed in IPA for 3 h. The sample was removed from IPA after 3 h and the surface of the samples was cautiously cleaned with tissue paper. Afterwards, the samples were quickly weighted and placed in an oven (Drying Oven, POL-EKO, Poland) for 2 h at 45 °C for the complete evaporation of IPA and the dry mat/yarn was weighed again.

The Fourier-transform infrared spectroscopy (FTIR) spectra of the sample mats were recorded on a Nicolet iS5 spectrometer (Thermo Fisher Scientific, Waltham, MA, USA) in the range of 600 to 4000 cm^−1^ by the ATR technique using germanium (Ge) crystal in the absorbance mode.

The mechanical properties of mats were verified using a tensile module equipped with a 20 N load cell (Kammrath Weiss GmbH, Germany). The samples, with a width of 4 mm, were mounted between clamps with a gap of 6 mm and were uniaxially stretched at an extension rate of 25 μm s^−1^. The tests for PS, and PS–EG were performed five times for each specimen. Stress–strain curves were prepared using OriginPro8 software. The Origin integrate function was used to calculate the maximum stress, strain at maximum stress, strain at break, and toughness. The thickness of specimens was measured from SEM images showing the cross-sectional view of samples.

The thermal properties of the electrospun samples were analyzed using differential scanning calorimetry (DSC, Mettler Toledo, Columbus, OH, USA). The samples were prepared by cutting pieces of each mat and sealing them into an Al crucible. The samples were heated from 25 to 325 °C with the rate of 10 K min^−1^ under 50 ml min^−1^ N_2_ purges. The STARe evaluation software was utilized to evaluate the *T*_g_ of the fiber mats. The DSC scan was performed 3 times for each sample.

The *λ* was measured *via* a FOX 50 heat flow meter (Laser Comp, USA) calibrated on the Pyrex glass standard. The value of the *λ* was calculated from 256 counts from the block, which have a stable value of heat flux. All tests were performed with the temperature gradient of 5 °C in the system, where the top plate was hotter than the down plate, which prevented natural convection from influencing the results.^103^ The presented results were calculated from the last 3 stable blocks, where the obtained measurement error was lower than 1%. In this test, fiber mats with a diameter of 60 mm were put into the measuring gap between the hot and cold plates with measured thicknesses of 5 mm for PS and PS–EG, respectively. The temperature of the top and down plates was measured by the set of thermocouples with a measuring surface with a diameter of 25 mm at the center of the plate. For the experiments, 15 layers of pristine PS and PS–EG electrospun mats were stacked together to fill the gap between the hot and cold plates.

The change in surface temperature of the prepared mats with time was analyzed using a thermal camera (FLIR T560, USA). The experimental setup is illustrated in the Fig. S6a (ESI†). For this experiment, the hot plate (TLC plate heater III, CAMAG, Switzerland) was heated from RT to 60 °C. The initial surface temperature of the 4 × 4 cm mat was varied from 24 to 26 °C. The mat's average surface temperature was measured from the middle section of each mat using the average box measurement tool in FLIR Tools software. The measurement was conducted three times per sample. The used emissivity and distance between the sample and the thermal camera were set at 0.96 and 40 cm, respectively. In the second thermal experiment, fiber mats were exposed to thermal radiation from an infrared (IR) lamp with a power output of 100 W. The mat was positioned vertical on a stand to allow for proper exposure to IR radiation (Fig. S6b, ESI†). The distance between the sample and the IR lamp and the thermal camera were 40 and 60 cm, respectively. During this time, the infrared radiation increased the temperature of the mat, simulating heat transfer through radiative means. After 5 minutes of heating, the IR lamp was turned off, and the mat was allowed to cool naturally in ambient air for an additional 5 minutes. This phase enabled the mat to release heat into the surrounding air, returning to a lower temperature.

The thermal performance of the yarn was carried out *via* a methodology as reported in the recent literature, where the resistive wire was utilized as a heating source.^4^ In brief, the electrospun yarn sample of 15 cm in length was heated up by applying current to the resistance wire (diameter of 200 μm), and the surface temperature of the yarns was captured by the thermal camera using a micro-lens. After heating the resistive wire-coated yarn sample for 10 min, the images of the fiber sample were acquired with 10 s time intervals. A reference tape (emissivity = 0.96, 3 M Scotch) was attached to the wires to ensure that all the resistance wires had a similar temperature. The average surface temperatures were calculated using average lines in FLIR Tools software from three different samples and from three different sections on the coated fiber. The infrared images were taken at *T* = 23 °C and RH = 25%.

## Author contributions

M. D., J. K.-K., U. S. conceived the presented idea. M. D., J. K.-K. prepared figures. J. K.-K. carried out the fiber mats preparation. M. D. and A. M. are responsible for yarns preparation. M. D., J. K.-K. and A. M. carried out the analysis of experimental data. W. P designed measurement of the thermal conductivity coefficient for fiber mats. M. D., J. K.-K. and U. S. wrote the manuscript. All authors reviewed the manuscript. U. S. supervised the project with funding acquisition.

## Data availability

Any additional data from this work are available from the corresponding author upon reasonable request.

## Conflicts of interest

The authors declare no conflict of interest.

## Supplementary Material

MA-006-D4MA01162G-s001
