# Supplementary material for: Enhanced thermal management of mats and yarns from polystyrene fibers through incorporation of exfoliated graphite
Source: Mater Adv. 2025 Feb 17;6(6):1859–68. doi: 10.1039/d4ma01162g (PMC11848512; doi:10.1039/d4ma01162g)
Supplement: MA-006-D4MA01162G-s001 [file MA-006-D4MA01162G-s001.pdf]

Supplementary Information (SI) for Materials Advances.
This journal is © The Royal Society of Chemistry 2025

Supporting Information to:

Enhanced Thermal Management of Mats and Yarns from Polystyrene Fibers Through Incorporation of Exfoliated Graphite

Madhurima Das[#a], Joanna Knapczyk-Korczak[#a], Ahmadreza Moradi[a], W. Pichór[b], Urszula Stachewicz[a*]

[a]Faculty of Metals Engineering and Industrial Computer Science, AGH University of Krakow, Krakow 30-059, Poland

[b]Faculty of Materials Science and Ceramics, AGH University of Krakow, al. A. Mickiewicza 30, 30-059 Kraków, Poland

<u>This Supporting Information include:</u>

Table S1. The mechanical properties obtained from stress-strain curves of electrospun mats.

Figure S1. The representative image of exfoliated graphite (EG) flakes.

Figure S2. a) Histograms with distribution curves representing the fiber diameters of PS and PS-EG in mats, and b) the average fiber diameter of PS and PS-EG fibers vs. the conductivity of the solution for electrospinning.

Figure S3. DSC diagram of PS, PS-EG mat, representing no melting peak under 25°C to 325°C temperature scan range.

Figure S4. Stress vs. strain curves of electrospun randomly oriented fibers mats: a) PS, and b) PS-EG.

Figure S5. SEM micrographs representing the surface morphology of: a) PS and b) PS-EG fibers in yarns. Histograms with distribution curves representing the distribution of: c) fiber diameters and d) bead diameters of PS and PS-EG fibers in yarns.

Figure S6. The experimental setup for heating the mats on a) the heating plate, and b) by infrared radiation.

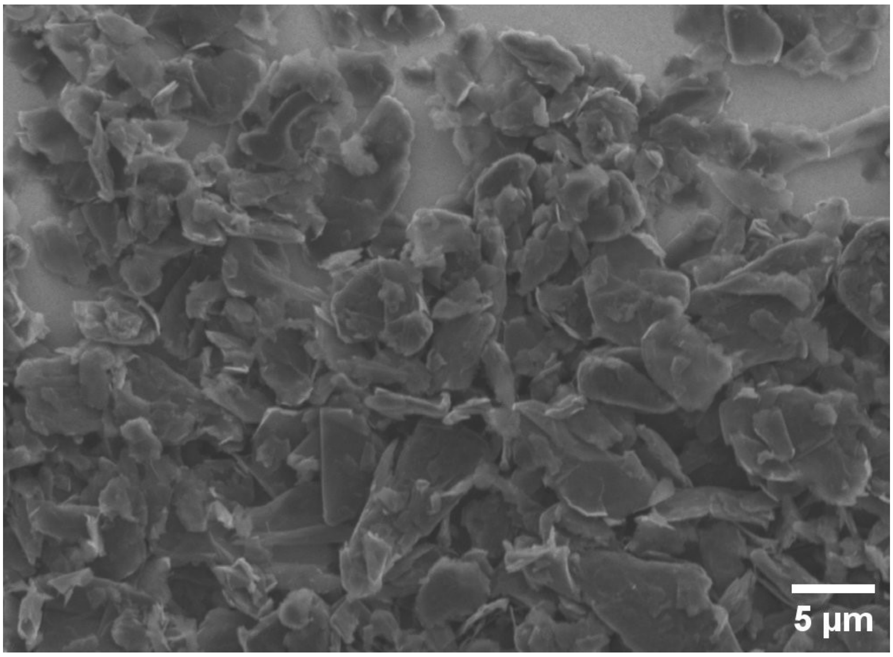

Figure S1. The representative image of exfoliated graphite (EG) flakes.

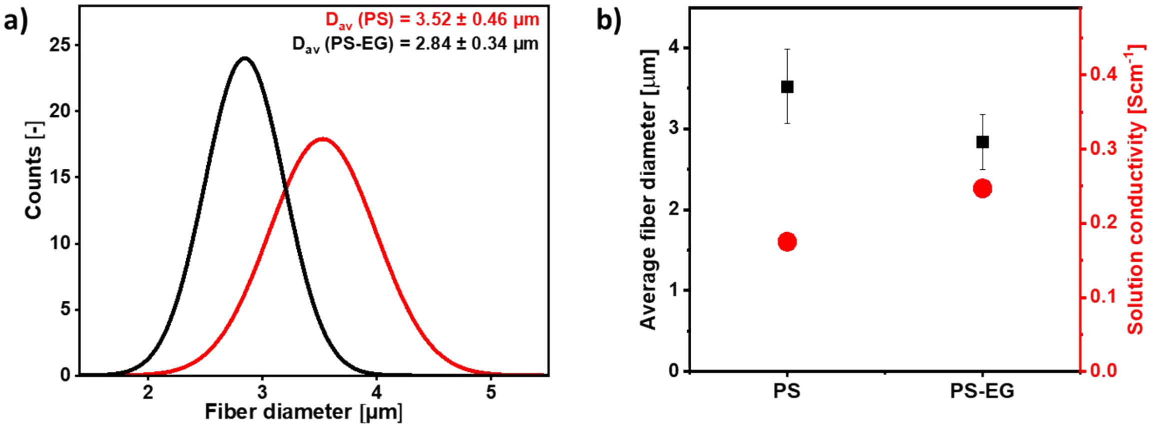

Figure S2. a) Histograms with distribution curves representing the fiber diameters of PS and PS-EG in mats, and b) the average fiber diameter of PS and PS-EG fibers vs. the conductivity of the solution for electrospinning.

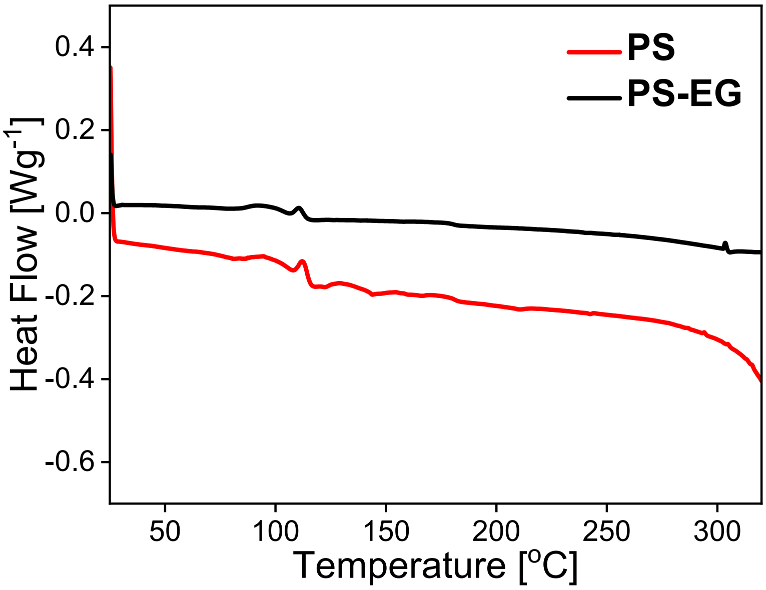

Figure S3. DSC diagram of PS, PS-EG mat, representing no melting peak under 25°C to 325°C temperature scan range.

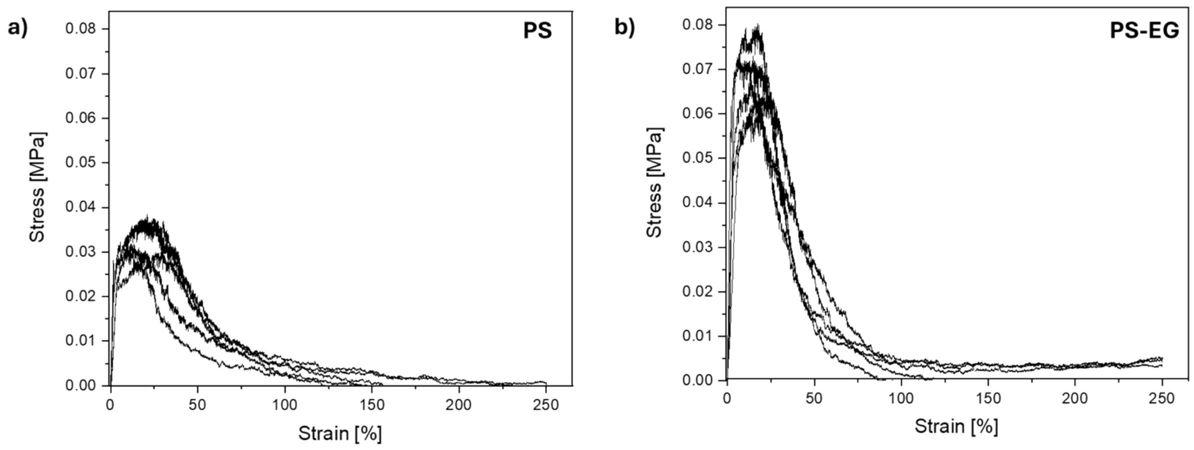

Figure S4. Stress vs. strain curves of electrospun randomly oriented fibers mats: a) PS, and b) PS-EG.

Table S1. The mechanical properties obtained from stress-strain curves of electrospun mats.

	PS	PS-EG
**Maximum stress (MPa)**	0.03 ± 0.01	0.07 ± 0.01
**Strain at max. stress (%)**	17.41 ± 10.20	15.75 ± 5.51
**Toughness ($MJ \cdot m^{-3}$)**	1.73 ± 0.31	2.93 ± 0.52

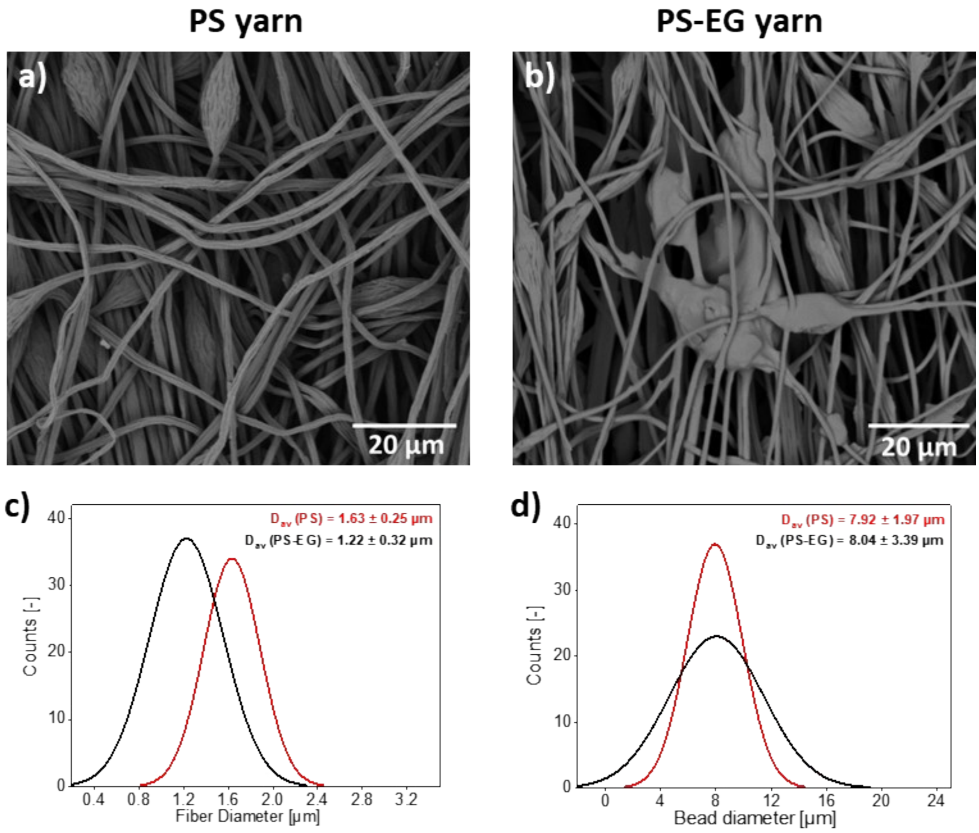

Figure S5. SEM micrographs representing the surface morphology of: a) PS and b) PS-EG fibers in yarns. Histograms with distribution curves representing the distribution of: c) fiber diameters and d) bead diameters of PS and PS-EG fibers in yarns.

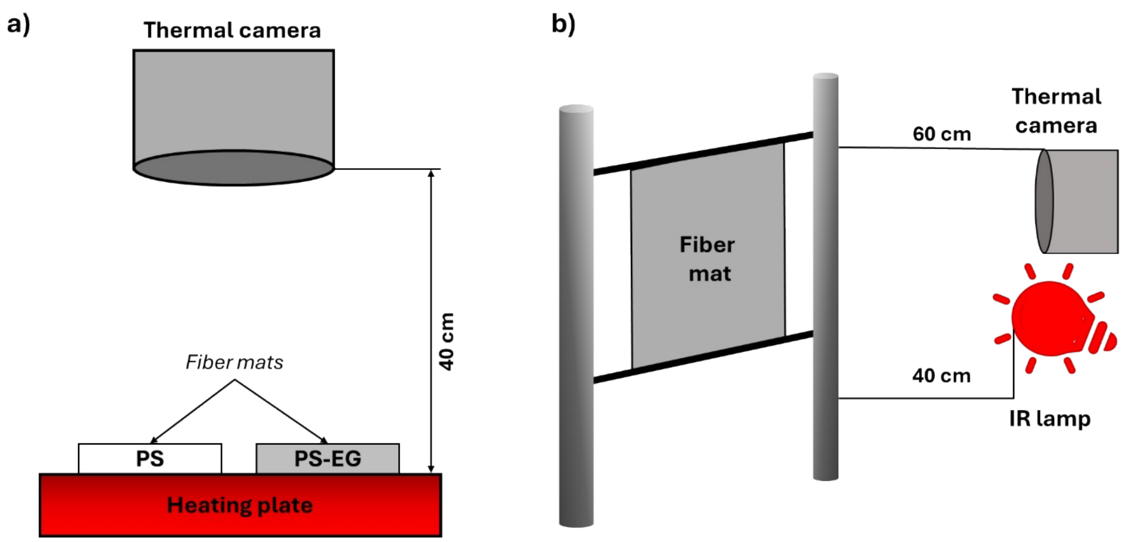

Figure S6. The experimental setup for heating the mats on a) the heating plate, and b) by infrared radiation.
